# Experiences of stigma among women living with HIV attending sexual and reproductive health services in Kenya: a qualitative study

**DOI:** 10.1186/1472-6963-14-412

**Published:** 2014-09-20

**Authors:** Manuela Colombini, Richard Mutemwa, Jackie Kivunaga, Lucy Stackpool Moore, Susannah H Mayhew, on behalf of the Integra Initiative

**Affiliations:** London School of Hygiene and Tropical Medicine, London, UK; Population Council, Nairobi, Kenya; AIDS 2014, Melbourne, Australia

**Keywords:** Stigma, Women living with HIV, Kenya, Integration, Sexual and reproductive health

## Abstract

**Background:**

Researchers have widely documented the pervasiveness of HIV stigma and discrimination, and its impact on people living with HIV. Only a few studies, however, have analysed the perceptions of women living with HIV accessing sexual and reproductive health (SRH) services. This study explores the experiences of stigma of HIV-positive clients attending family planning and post-natal services and implications for service use and antiretroviral therapy (ART) adherence. Our aim was to gain a better understanding of the impact of various dimensions of stigma on service use and ART adherence among HIV clients in order to inform the response of integrated SRH services.

**Methods:**

In-depth interviews were conducted with 48 women living with HIV attending SRH services in two districts in Kenya. Data were coded using Nvivo 8 and analysed using a thematic analysis approach.

**Results:**

Findings show that many women living with HIV report high levels of anticipated stigma, resulting in a desire to hide their status from family and friends for fear of being discriminated against. Many women feared desertion following disclosure of their positive status to partners. Consequently some women preferred to hide their status and adhere to HIV treatment in secret. However, the majority of study participants attending postnatal care (PNC) services also revealed that anticipated stigma does not adversely affect their HIV drug uptake and ART adherence, as their drive to live outweighs their fear of stigma. Our findings also seem to suggest a preference for specialist HIV services by some family planning (FP) clients because of better confidentiality and reduced opportunities for unwanted disclosure that could lead to stigma.

**Conclusions:**

The findings highlight that anticipated stigma leading to low disclosure is widespread and sometimes reinforced by health providers’ actions and facility layout (contributing to enacted stigma). However, the motivation to stay healthy and look after the children appears in many cases to override fears of stigma related to ART adherence in our client-based sample.

## Background

In sub-Saharan Africa, women remain disproportionately affected by the HIV epidemic, accounting for 58% of all people living with HIV in the region [1].

Researchers have widely documented the pervasiveness of HIV stigma and discrimination at community level, and its impact on accessing HIV prevention and treatment [2], and care and support services [3]. It can also create barriers to the use of condoms and uptake of HIV testing and prevention of mother to child transmission programs (PMTCT) as well as other services (such as legal, employment, psychosocial care) [4, 5]. A study in rural Kenya suggests that HIV-related stigma is associated with a low rate of delivery by skilled attendants [6].

Existing theoretical research on HIV-related stigma also highlights how various aspects of stigma negatively impact on healthcare access, and health outcomes [7, 8], and is associated with stress, depression, and lower perceived quality of life among people living with HIV [9]. Different types of stigma have been differentiated as enacted stigma (actions resulting from stigma, also known as discrimination), anticipated stigma (fear that stigma will be experienced) and internalised stigma (the internalisation of the negative or devaluing attitudes) [10–15]. HIV-related stigma and discrimination can manifest themselves in different ways: through prejudice, negative attitudes, abuse and maltreatment directed at people living with HIV.

Several studies have analysed the perceptions of stigma among women living with HIV (WLWH) accessing antenatal and PMTCT services [16–19], though little has been documented about experiences of stigma by family planning (FP) and post-natal care (PNC) clients and its service implications. This study sought to explore the perceptions of stigma among clients living with HIV attending FP and PNC services in Kenya. Our aim was to gain a better understanding of the implications of various dimensions of stigma on service use, breastfeeding practices and ART adherence among HIV clients in order to inform the response of integrated SRH services.

## Methods

### Sites

This study was conducted with women attending nineteen public health facilities, including 1 provincial hospital, 8 district hospitals, 6 sub-district hospitals and 4 health centres from urban and rural areas of Central and Eastern Provinces in Kenya. Nine of these facilities offer integrated on-site HIV and PNC services: mostly HIV counselling and testing and antiretroviral therapy (ART) referral at MCH. After six months post-partum, most women are transferred (from PNC) to HIV clinics for their own HIV treatment and care. Eight facilities offer integrated HIV and FP services: HIV/STI counselling and testing services in the same FP consultation room, and referral to on site Comprehensive Care Centres (CCCs – specific HIV clinics) for specialised management.

Central and Eastern provinces have HIV prevalence levels in women age 15-49 between 3% and 5% overall, except North Eastern province where the prevalence is about 1 percent [20].

Ethical approval for this study was obtained from the Kenya Medical Research Institute (approval nos. SCC/113 and SCC/114), from the London School of Hygiene and Tropical Medicine (approval no. 5426), and from the Population Council Institutional review Board (approval nos. 443 and 444).

### Participants and procedures

This articles draws on a total of 48 qualitative in-depth interviews (IDIs) with HIV-positive women. Semi-structured interviews were conducted with women living with HIV attending SRH services in Eastern and Central Provinces. The sample – originally recruited from health facilities - was randomly selected from a larger cohort of HIV-positive women who participated in a study on integration of family planning and HIV services in Kenya and Swaziland [21]. In order to maintain geographical representation (to gather information across sites in the 2 Provinces), a sample of women was selected from nineteen study facilities. Face-to-face interviews were conducted in Kiswahili by 8 trained local interviewers between January and March 2011. IDIs were conducted in private locations preferred by the interviewees, primarily homes. No interviews took place in health facilities. Written informed consent was obtained from all participants before they were interviewed. In-depth interviews were designed to take approximately an hour to complete. All interviews were audio-recorded.

### Interview guides

A semi-structured topic guide was designed to elicit information on women’s experiences of SRH services they visited (and from where they were recruited), and their views and perceptions of stigma at health facility and community levels. The final guide was comprised of nine main parts with opening questions and probes for each section to help explore issues in-depth. Main topics - originally pre-determined based on the study objectives and on the quantitative findings we wanted to explore in-depth - covered included stigma, process and outcomes of disclosure, adherence and potential fear of disclosure; breastfeeding practices and challenges; discrimination by health providers.

### Data analysis

Audiotapes of the interviews were transcribed in Kiswahili by the local interviewers and translated to English. Data were analysed using a thematic analysis approach [22]. Data were coded using the Nvivo 8 software. Initial coding of the transcripts was conducted by the first and last authors according to major topics from the interview guides, but new codes and themes were also developed based on the interviews’ data. Quotations, relevant passages and memos regarding each topic were brought together into matrices, organised into categories and sub-categories, and analysed to identify common themes and variant views. The hierarchy of codes was constantly adjusted and refined during the analysis process, until fewer broad overarching themes were identified. Measures to ensure validity of the results included discussion of preliminary results with local partners working in SRH in Kenya.

## Results

Table 1 presents the socio-demographic characteristics of the 48 study participants. We explored how stigma was perceived and experienced by WLWH and what the main effects of this seemed to be. This section is divided into two main parts: first, women’s experiences of stigma at health service level; second, the effects of stigma on low disclosure of HIV status and specifically on, breastfeeding and ART adherence as two behaviors that are significantly affected by lack of disclosure.

**Table 1:** **Socio-demographic characteristics of the study participants**

Characteristic	N = 48: n (%)
**Age:**	
*18-24*	4 (8)
*25-34*	32 (67)
*35 & over*	12 (25)
**Marital status:**	
*single*	13 (27)
*married-monogamous*	31 (65)
*married-polygamous*	4 (8)
**Educational attainment:**	
*none*	0 (0)
*primary*	35 (73)
*secondary*	13 (27)
*college*	0 (0)
**Employment status:**	
*unemployed*	19 (40)
*self-employed*	22 (46)
*employed*	7 (14)
**Breadwinner in household:**	
*myself*	14 (29)
*partner*	30 (63)
*both (of us)*	0 (0)
*other*	4 (8)
**Number of living children:**	
*0 =*	1 (2)
*1 =*	5 (10)
*2 =*	14 (29)
*3 =*	12 (25)
*4 =*	8 (17)
*5 & over =*	8 (17)

The majority of women in our study reported anticipated stigma, while only a few reported enacted stigma. There were few differences between FP and PNC clients (where they exist they are reported), primarily linked to breastfeeding dilemmas and ART adherence.

### Experiences of stigma at health facility level

The majority of women interviewed reported anticipated stigma. Most of the study clinics observed offering integrated FP and HIV services have audio-privacy during consultations, and women were seen alone and asked questions in complete privacy. However, some respondents attending family planning services stated that they feared people would discover their status just by attending the facility’s services. Some mentioned they did not feel free because the physical space of the hospital’s clinic was too open, thus leading to lack of privacy and potential involuntary disclosure.

“I: *And do you feel comfortable here at the clinic?*

*R: They have exposed us so much…[…] The place is too open.[…]: Everybody…when they come in, they see us. At least we would like somewhere hidden....I don't feel free*.” [FP 010476]

On the other hand, one FP client said she chose her clinic because nobody knew her: *‘because I’m not known there so much’* [FP 06 08 02].

One PNC client also mentioned that she would rather travel further than to be transferred to another hospital, because she liked the doctors in her [HIV] clinic and she feels ‘free’.

*“R: those doctors, those who treat us at X [name of community/clinic]* [are friendly]*, which is why when another day they told me they would give transfer to Y [name of community/clinic] I told them I would rather pay that fare* [higher fare to go to X clinic] *and go to X [name of community/clinic]. They are friendly, and they give you the chance to ask the questions that you want. There you are free.”* [PNC 140433]

Interestingly, lack of trust in maintaining confidentiality by the health providers was only mentioned as a challenge by some FP clients, and not by PNC ones. One woman said she would prefer to have one single provider treating her because she would fear others may “spread” her status (as she would have to disclose it to new health providers every time they change).

*“I: the reason for being attended by that one provider?*

*R: Because it’s him/her who understands me, yes. It’s him/her who knows my status. If I am sent to someone else….to another doctor* [who does not know my status] *I am scared to say, to tell him/her… because, I see as if he/she may spread* [the information about my status].” [FP 060802]

Changing of providers for every service was also said to have an impact on clients’ disclosure.

*“I would prefer that a family planning provider remains [offering] family planning every day you meet him/her, because you’ll be able to explain your problem. […] But if you find a different person* [every time you go for FP] *you will wonder ‘now where will I start to explain to this one’. […] Maybe someone is afraid. You know, you can’t tell someone private issues when you have just met. Maybe, if you are free with each other… that is why I am saying: if you meet him* [provider] *today and meet him again next time, maybe you will be free* [to talk] *to each other and you will be able to tell them.”* [FP 11117]

Some FP clients said it was good to be separate from ‘other people’ [not infected] because there was no fear of involuntary disclosure and therefore they felt free; and found mutual support from others because they were all positive.

*“While inside* [the CCC: comprehensive care centre] *we feel free…because not many people know what kind of people these are. It is not the way it was there… [..] The time I was in the hospital, I had fears and that is why some people did not want to be identified. Because when someone sees you* [there at the clinic], *they say that that person is sick. But on this side we know each other… We think the separation is good, even when you tell someone they don’t fear, there’s no secrecy because you are telling them, all of us here are with one* [same] *condition”.* [FP 041649]

Although the PNC clients interviewed had not expressed a clear preference for separated services, one woman mentioned the benefits of being able to ‘exchange ideas’ and advice with other positive women. Interestingly, many of the PNC clients started their HIV services (PMTCT) at ANC and PMTCT clinic.

Only one PNC client reported an experience of enacted stigma at a health facility: just after delivering her baby she experienced involuntary disclosure due to lack of protection of confidentiality while at the facility. We consider lack of confidentiality/privacy leading to unwanted disclosure as a form of enacted stigma.

“*R: I had to tell her [mother in law], she is the one who took me to the hospital and she was there when I was giving birth. She knows that a woman is supposed to breastfeed after she has given birth, something she never saw happening because I was told not to breastfeed the baby and saw the service provider administering some drugs to the baby and she could not understand what was happening and therefore I had to tell her the truth*.”[PNC 011463]

### Effects of stigma on low disclosure

One of the main emerging themes was how stigma influenced low disclosure among WLWH. In our study of stigma ‘fears’ were also a key factor influencing women’s behavior on disclosure: fear of discrimination.

More than half of the women in the study disclosed their HIV-positive status only to their close family (mother, sister or father) for fear of being discriminated against because of negative community beliefs about people living with HIV. Anticipated stigma following disclosure was quite prevalent among all women in the study. Many said they were afraid to disclose their status because they feared others would start gossiping about them.

*“R: I am afraid of the others, you can tell someone then he starts talking badly, begin to go tell and tell others”.* [PNC 131530]

Some women who reported not having disclosed their status said that they feared being blamed by relatives for having transmitted HIV to the partner even if he was the one who was infected first.

“*I: So why have you not told him?*

*R: Now it is that fear. I do not have a way of explaining to him.*

*I: Ehe? What are you afraid of?*

*R: t … that his parents know me, and they do not know about my status, they may say: ‘Our son was infected by that girl’, I do not know what… Mm. And it may be possible that he is the one who … infected me. No one knows who infected the other”.* [PNC 140433]

Over a fifth of the respondents cited a certain degree of fear of partner’s rejection and/or negative reactions. In particular, five women cited the fear that someone else could tell their husbands they are ill and, as a consequence, the husband might either leave them or abuse them.

*“I: And why haven’t you told anyone else?*

*R: They* [people] *can do bad things to you […]. They can tell my husband. […] If my husband got to know about it* [that she is positive] *he will be mad with me and quarrel […] He can tell me to go back home so that [we] separate. I don’t want that to happen”.* [PNC 027126]

For some of the women who disclosed to their husband or partner, these fears of abandonment became real.

*“I: How did he know?*

*R: I told him. When I told him, he ran away from me”.* [FP 050435]

Although there were no reports of physical violence following disclosure of HIV-positive status, four reported that the husbands had initial negative verbal outbursts and blamed them for bringing the “disease” home.

*“He abuses me, he says he will look for another wife […] he did not beat me”.* [FP 010247]

All of the women who had disclosed their status stated they feared disclosure and its potential consequences. Among the reasons stated for disclosing to only one or very few people were: fear of being rejected, isolated and not helped; fear of being blamed; fear that people may disclose their status to others.

*“R: I told them* [family] *only recently.*

*I: Okay. . . Who did you tell first?*

*R: My mother […] I felt bad. […] I thought they will discriminate me take… me negatively […] I mean . . . they will stop helping me. . .”.* [PNC 140357]

Nevertheless disclosure was also found to have positive effects, particularly among PNC clients. Among the women who disclosed their status, nearly a third cited a positive outcome following disclosure to their husband/partner, especially if both partners were HIV-positive. Couple counseling and testing and condom use were most often mentioned.

*“I told my partner so that he also knows his status* [being advised by doctor to disclose to husband and get couple VCT]*. As a result, my husband went and he was found positive.”* [PNC 140624]

### Stigma and breastfeeding among PNC clients

In the past few years, Kenyan clinical practices seem to have given mixed messages to both health workers and WLWH about breastfeeding. As a result, educational messages highlighting the positive health benefits of exclusive breastfeeding have not fully reached the health providers and the women on the ground.

This section focuses primarily on PNC women. FP clients were not asked about breastfeeding directly. Several PNC women reported that anticipated stigma (and the fear of involuntary disclosure) affected their breastfeeding practices. All the PNC respondents said they received clinical advice on exclusive breastfeeding for at least 6 months. Only 2 women were advised not to breastfeed the baby and use formula milk instead. Although some women managed to breastfeed their baby for at least 6 months, it seems that despite the advice received, many PNC women breastfed for only few weeks or months and then stopped because they were still afraid of transmitting the virus to their baby, or because they got sick. Several respondents mentioned they feared that others, such as neighbours or relatives, may gossip about them if they knew they were not breastfeeding their baby. A few reported that people would check whether a woman was breastfeeding or not in order to detect someone’s status.

*“I hear many gossiping and saying “so and so had the virus and is giving birth. Why is she giving birth?” They even follow her to the house to see if she is breastfeeding. If they find she is not breastfeeding they go away and say she has the virus. […] they are saying she is giving the virus to the children”.* [PNC 070222[1]]

For these reasons, some women said they hide the fact that they were not breastfeeding because they feared people would think they were positive.

*“Even at this moment no one knows that I don’t breastfeed.[laughs] You just know how people talk? They start gossiping about you. Even if they knew, I did not want to show them myself.”* [PNC 131547]

One respondent also mentioned how she feared people would think less of her if she revealed she stopped breastfeeding her baby.

“*I: why have you kept it a secret to them that you are not breastfeeding your child?*

*R: I do not want them to know …..If they do…will go around saying things here and there…* [They] *will see me as a foolish person…. They will be seeing me as a person of no importance*”. [PNC 0105124]

Even women who were still breastfeeding reported some fear of being stigmatised by the community if they stopped breastfeeding.

“*I won’t feel good. They can hate you, they can gossip about me, they start talking about me badly* [if they know I stopped breastfeeding].” [PNC 140448]

Only one woman reported how the breastfeeding advice she got at the clinic helped her focus on her individual choice rather than being influenced by any external factors. This also helped her overcome her fear of stigma.

“*I would not feel discriminated if I do not breastfeed my baby*… *because we were attending the teachings* [at the MCH clinic]*, they* [the nurses] *emphasized to us that ‘this time, it is your responsibility, you can choose for yourself the feeding method you want to use to raise your baby with’…these are the things I can tell others* [members of community who question her breastfeeding practices]*’.* [PNC 081105]

### Stigma, secrecy and ART adherence

This section reports findings primarily for PNC clients, as the majority of FP clients were not directly asked about adherence and/or did not reveal whether or not they were on antiretrovirals (ARVs).

No reports of enacted stigma related to drug uptake emerged from the findings. The majority of women interviewed mentioned experiences of anticipated stigma. Of the fifteen PNC respondents who said they were on ARVs only two women said they take their ARVs in secret because they have not disclosed their positive status and they fear that people and/or their husband would realise they are ill if they are seen taking pills.

“*I: You told me that your husband has no knowledge that you are using these drugs? How do you take these drugs?*

*R: I put them in my pocket. […] He doesn’t come checking my pocket […] I wait for him to go”.* [PNC 0207126]

Others said they were sometimes afraid to take their medications for fear of others finding out their status.

*“R: When there are people* [it is hard to take ARVs] *because I have not told them. Now if they see me taking medication? They will ask why I am taking medication; they will ask why I am taking medications … like when I had not told my husband that I wasn’t even taking them…because I was hiding from him so he could not to see them.”* [PNC 070222]

Although we do not have information on actual adherence, most of the women reported that they were taking their ARVs even if they did not disclose their status and showed high motivation for adherence. One woman said that she would always find a way to take her pills even if she did not tell her husband.

*“I: Earlier you told me no one knows you are using these drugs. If your husband does not leave, would you take the medicine?*

*R: He cannot make me not take my medicine. I will hide and take it. … I will always find my way out”.* [PNC 0207126]

Many said that ARVs made them live longer and feel better and they can take care of their children.

*“You know if you use those medications, it helps you to live longer. […] If you don’t use them, you will not fare* [feel] *well. […] I felt it was proper to use so that I can take care of my children”.* [PNC 091237]

## Discussion

The article explored how stigma was perceived and experienced by women living with HIV and what the main effects of this seemed to be on their lives. Only a few women in the study reported experiences of enacted stigma, primarily being abandoned by husbands. Only one report was mentioned of enacted health facility stigma. The low disclosure of experiences of actual discrimination could be the result of courtesy bias, where the respondents, who were originally recruited in health facilities, may perceive the interviewers as part of the medical team. However, as we did not interview them at the facilities, but at a place of their choice (usually home) and they had already taken part in a survey questionnaire, we believe that such bias was minimised.

The main type of stigma evident among our respondents was anticipated stigma from community and family members, particularly husbands or partners. Some differences appeared between FP and PNC clients in relation to reported preferences for integrated services by FP clients, reported adherence levels among PNC women, and breastfeeding dilemmas experienced by PNC clients.

The study suggests that fear of stigma (i.e. “anticipated stigma”) is widespread among the women interviewed. This fear has several important effects that can lead to behavioral consequences, such as poor disclosure, unsafe breastfeeding practices, and reduced ART adherence or making it more stressful [23, 24]. Nevertheless, our findings suggest that the motivation to adhere to drug regimens to protect children and preserve health overrode the fear of stigma for most of our respondents attending PNC services. It is an interesting result that seems to contradict existing literature showing a link between internalised stigma and ART adherence [25, 26]. This may be due to the fact that this is a specific sample of women with young children and who are regularly attending SRH services.

Our data show how a number of women feared that if they disclosed their status they would face negative outcomes from their partners/husbands or significant others, including abandonment, separation, verbal abuse, loneliness and loss of financial security. These findings are consistent with other recent research linking HIV stigma to low disclosure [27–32]. Some of these fears of separation (and its economic related consequences) and violence were grounded in reality, as shown by the actual outcomes of stigma described in our study.

Early cessation of breastfeeding and mixed feeding could increase the risk of HIV transmission to infants, as recent evidence shows [33]. Recently revised WHO recommendations are that breastfeeding exclusively for six months does not increase HIV transmission risk and is safer for infant health than mixed feeding or formula feeding in situations where formula feeding is not “acceptable, feasible, affordable, sustainable, and safe” [34]. Our findings suggest that postnatal women’s fear of stigma due to involuntary disclosure has an impact on feeding preferences, often resulting in continuation of breastfeeding (despite their fear of HIV transmission to the baby) and stress caused by feeling the need to hide their status (particularly in relation to mixed feeding, as the mother-in-law may give solids or water to the baby before the 6 months threshold). Other studies have also highlighted how socio-cultural influences impact on infant feeding decisions among women living with HIV [19, 35–39]. Stigma and inadequate knowledge about safe infant feeding are pushing women into not following the WHO recommendations on exclusive breastfeeding. The Kenyan guidelines for PMTCT recommend exclusive breastfeeding for the first 6 months and then to continue breastfeeding up to 1 year with appropriate complementary feeds [40]. However, this advice, which could help reduce stigma dramatically, has not fully reached the nurses – and the women - on the ground.

More should be done within healthcare facilities to ensure that WLWH receive clear information on how exclusive breastfeeding may achieve lower risks of transmission. If some of the women interviewed had continued breastfeeding as recommended, they would not have faced the stress of stigma (or fear of stigma) from the community.

Some women acknowledged that openly taking their HIV drugs is a potential risk for involuntary disclosure, which has also been recognised in other African studies [41–43]. However, the majority of study participants attending PNC services reported that anticipated stigma does not adversely affect their HIV drug uptake and ART adherence. Women’s strong motivation (especially if they were mothers of young children) to adhere to drugs and stay healthy for their children appears to outweigh their fear of stigma. Although this finding could not be generalised to all other WLWH, ours being a selected sample of people who regularly attend HIV clinics, it still seems an important topic to explore further. We could only find one other regional study reporting that the maternal desire to protect the child is the main drive for drug uptake among positive pregnant women [44]. As stigma is detrimental to women’s emotional and mental health, further improving it is a crucial (but neglected) part of achieving better health outcomes for WLWH. Addressing anticipated stigma, for example through dealing with messaging on safe breastfeeding and promoting positive support networks, would be a big contribution to this.

Our study shows that many women attending PNC services feared stigmatisation at a health facility. In particular, the lack of privacy and confidentiality - and limited trust in service providers’ ability to maintain confidentiality - increases fear of stigma among WLWH attending health services. Fear of unwanted disclosure can be reinforced by the providers’ acts and by the health facility settings, as shown in other recent studies [45–47]. Our data also seem to reveal that having one provider offering integrated services could reduce the fear of repeated disclosure and improve respect of confidentiality. Our data suggest that counselling on risks of disclosure and how to cope with HIV- related stigma is scarce within the selected health facilities. A study with women attending ANC services in Uganda shows how women living with HIV felt they did not receive sufficient counselling after HIV testing to address their fears and how to cope with a positive diagnosis [31]. Health care staff must be sensitive to the fears and concerns of the HIV-positive mother about their infection, and issues of disclosure to her partner. Women should be more supported and receive counselling on the pros and cons of disclosure (i.e. a balanced and non-judgemental view) rather than being manipulated or pressured into disclosing, and their choice of disclosure (if, when, how and to whom) should be fully respected.

It is usually argued that integrated services are more anonymous and confidential than having to be seen going to a separate HIV clinic and thus less stigmatizing [48–50]. However, our findings seem to suggest a preference for separate HIV services by some FP clients as they appreciated the freedom to talk and the social support in HIV clinics, and felt these had better confidentiality from providers and reduced opportunities for unwanted disclosure that could lead to stigma. This finding contributes to a small literature supporting this view from other studies in Swaziland and Zambia [47, 51].

The study results support the importance of strengthening health education sessions on safe breastfeeding practices and of integrating psychosocial support services for HIV-positive women – whether on site or off-site – into existing SRH and HIV services. The newly adopted Kenyan Minimum Package for Reproductive Health (RH) and HIV Integrated Services incorporates psychosocial support as an essential element of its package [52]. Providers at maternal health and child health (MCH) and Family planning (FP) units should be able to refer HIV-positive women to psychosocial support that could help reduce anticipated and internalized stigma. Moreover, the friendliness of health professionals and making clients feel they have the freedom to talk and ask questions has been proven to be an important characteristic for client satisfaction, and should not be lost when integrating HIV and PNC or FP services.

### Limitations

Being a qualitative study with a relatively small sample it is not possible to generalize the findings. It should also be acknowledged that these findings are based on what the women interviewed chose to report to the interviewer, and we do not have data about their actual adherence or breastfeeding practices. Moreover, this is a group of women attending health services and thus their responses may not be reflective of all women of reproductive age living with HIV.

## Conclusions

Many women in our sample reported anticipated stigma, although only a minority experienced enacted stigma. Anticipated stigma was found to be exacerbated by the actions of providers and by a lack of accurate or consistent information on safe breastfeeding practices. Anticipated as well as enacted stigma can lead to low disclosure, low ART adherence and unsafe breastfeeding practices. It is therefore important to tackle anticipated stigma, including through improving accuracy of information on safe-breastfeeding and improved confidentiality of clients using services. In particular some respondents noted that having a single provider (with whom trust is built over time) could decrease stigma and fear of involuntary disclosure. A number of women indicated preferences for specialist HIV sites for multiple service provision and this should be explored in further research.

Despite women’s fears of stigma we also found an overwhelming drive to adhere to their drugs in order to remain healthy and look after their children. This is a powerful motivation that should be built on through messaging on safe breastfeeding and promoting positive support networks.

## References

[1] **Regional Factsheet** 2012. http://www.unaids.org/en/media/unaids/contentassets/documents/epidemiology/2012/gr2012/2012_FS_regional_ssa_en.pdf

[2] Mahajan AP, Sayles JN, Patel VA, Remien RH, Sawires SR, Ortiz DJ, Szekeres G, Coates TJ (2008). Stigma in the HIV/AIDS epidemic: a review of the literature and recommendations for the way forward. Aids.

[3] UNAIDS (2012). Key Programmes to Reduce Stigma and Discrimination and Increase Access to Justice in National HIV Responses. Guidance Note.

[4] Genberg BL, Hlavka Z, Konda KA, Maman S, Chariyalertsak S, Chingono A, Mbwambo J, Modiba P, Van Rooyen H, Celentano DD (2009). A comparison of HIV/AIDS-related stigma in four countries: negative attitudes and perceived acts of discrimination towards people living with HIV/AIDS. Social Science and Medicine.

[5] Maman S, Abler L, Parker L, Lane T, Chirowodza A, Ntogwisangu J, Srirak N, Modiba P, Murima O, Fritz K (2009). A comparison of HIV stigma and discrimination in five international sites: the influence of care and treatment resources in high prevalence settings. Social Science and Medicine.

[6] Turan JM, Hatcher AH, Medema-Wijnveen J, Onono M, Miller S, Bukusi EA, Turan B, Cohen CR (2012). The role of HIV-related stigma in utilization of skilled childbirth services in rural Kenya: a prospective mixed-methods study. PLoS Med.

[7] Maluwa M, Aggelton P, Parker R (2002). HIV- and AIDS-related stigma, discrimination, and human rights. Health Hum Rights.

[8] Pinel E (1999). Stigma consciousness: the psychological legacy of social stereotypes. J Pers Soc Psychol.

[9] Simbayi L, Kalichman L, Strebel A, Cloete A, Henda N, Mqeketo A (2007). Internalized stigma, discrimination, and depression among men and women living with HIV/AIDS in Cape Town, South Africa. Soc Sci Med.

[10] Zelaya C, Sivaram S, Johnson S, Srikrishnan A, Suniti S, Celentano D (2012). Measurement of self, experienced, and perceived HIV/AIDS stigma using parallel scales in Chennai. India AIDS Care.

[11] Visser M, Kernshaw T, Makin JD, Forsyth B (2008). Development of parallel scales to measure HIV-related stigma. AIDS Behaviour.

[12] Holzemer WL, Makoae LN, Greeff M, Dlamini PS, Kohi TW, Chirwa ML, Naidoo JR, Durrheim K, Cuca Y, Uys YR (2009). Measuring HIV stigma for PLHAs and nurses over time in five African countries. SAHARA J.

[13] Holzemer WL, Uys L, Makoae L, Stewart A, Phetlhu R, Dlamini PS, Greeff M, Kohi TW, Chirwa M, Cuca Y, Joanne N (2007). A conceptual model of HIV/AIDS stigma from five African countries. J Adv Nurs.

[14] Simbayi L, Kalichman S, Strebel A, Cloete A, Henda N, Mqeketo A (2007). Internalized stigma, discrimination, and depression among men and women living with HIV/AIDS in Cape Town. South Africa Social Science & Medicine.

[15] Tsai AC, Bangsberg DR, Frongillo EA, Hunt PW, Muzoora C, Martin JN (2012). Food insecurity, depression and the modifying role of social support among people living with HIV/AIDS in rural Uganda. Social Science and Medicine.

[16] Turan JM, Bukusi EA, Onono M, Holzemer WL, Miller S, Cohen CR (2011). HIV/AIDS stigma and refusal of HIV testing among pregnant women in rural Kenya: results from the MAMAS Study. AIDS Behav.

[17] Bwirire LD, Fitzgerald M, Zachariah R, Chikafa V, Massaquoi M, Moens M, Kamoto K, Schouten EJ (2008). Reasons for loss to follow-up among mothers registered in a prevention-of-mother-to-child transmission program in rural Malawi. Trans R Soc Trop Med Hyg.

[18] Johnson K, Akwara PA (2009). Access to and Utilization of Antenatal HIV Counseling and Testing Services in Countries Heavily Affected by AIDS: who is Offered the Test, who Accepts the Test, and What is the Effect of Policy on Testing Uptake?. XXVI IUSSP International Population Conference.

[19] Turan J, Nyblade L (2013). HIV-related stigma as a barrier to achievement of global PMTCT and maternal health goals: a review of the evidence. AIDS Behav.

[20] Kenya National Bureau of Statistics (KNBS), ICF Macro (2010). Kenya Demographic and Health Survey. 2008-2009.

[21] Warren C, Mayhew S, Vassall A, Kimani J, Church K, Obure C, du-Preez N, Abuya T, Mutemwa R, Colombini M (2012). Study protocol for the integra initiative to assess the benefits and costs of integrating sexual and reproductive health and HIV services in Kenya and Swaziland. BMC Public Health.

[22] Taylor SJ, Bogdan R (1984). Introduction to Qualitative Research Methods: The Search for Meanings.

[23] Quinn DM, Chaudoir SR (2009). Living with a concealable stigmatized identity: the impact of anticipated stigma, centrality, salience, and cultural stigma on psychological distress and health. J Pers Soc Psychol.

[24] Earnshaw V, Chaudoir S (2009). From conceptualizing to measuring HIV stigma: a review of HIV stigma mechanism measures. AIDS Behav.

[25] Izugbara CO, Wekesa E (2011). Beliefs and practices about antiretroviral medication: a study of poor urban Kenyans living with HIV/AIDS. Sociol Health Illn.

[26] Katz IT, Ryu AE, Onuegbu AG, Psaros C, Weiser SD, Bangsberg DR, Tsai AC (2013). Impact of HIV-related stigma on treatment adherence: systematic review and meta-synthesis. J Int AIDS Soc.

[27] Simbayi LC, Kalichman SC, Strebel A, Cloete A, Henda N, Mqeketo A (2007). Disclosure of HIV status to sex partners and sexual risk behaviours among HIV-positive men and women, Cape Town, South Africa. Sex Transm Infect.

[28] Vu L, Andrinopoulos K, Mathews C, Chopra M, Kendall C, Eisele T (2012). Disclosure of HIV status to sex partners among HIV-infected men and women in Cape Town. South Africa AIDS and Behavior.

[29] Duff P, Kipp W, Wild TC, Rubaale T, Okech-Ojony J (2010). Barriers to accessing highly active antiretroviral therapy by HIV-positive women attending an antenatal clinic in a regional hospital in Western Uganda. J Int AIDS Soc.

[30] Awiti Ujiji O, Ekstrom AM, Ilako F, Indalo D, Wamalwa D, Rubenson B (2011). Reasoning and deciding PMTCT-adherence during pregnancy among women living with HIV in Kenya. Cult Health Sex.

[31] Rujumba J, Neema S, Byamugisha R, Tylleskar T, Tumwine JK, Heggenhougen HK (2012). “Telling my husband I have HIV is too heavy to come out of my mouth”: pregnant women’s disclosure experiences and support needs following antenatal HIV testing in eastern Uganda. J Int AIDS Soc.

[32] Medley A, Garcia-Moreno C, McGill S, Maman S (2004). Rates, barriers and outcomes of HIV serostatus disclosure among women in developing countries: implications for prevention of mother-to-child transmission programmes. Bull World Health Organ.

[33] Sturt AS, Dokubo EK, Sint TT (2010). Antiretroviral therapy (ART) for treating HIV infection in ART-eligible pregnant women. Cochrane Database Systematic Review.

[34] WHO, UNICEF, UNFPA (2010). Guidelines on HIV and Infant Feeding 2010: Principles and recommendations for infant feeding in the context of HIV and a summary of evidence 2010.

[35] Muhumuza M, Kizito N, Malende S, Masereka C, Tusiime W (2012). Factors that influence infant feeding options among HIV-positive mothers in Western Uganda. Exchange on HIV / AIDS Sexuality and Gender.

[36] Thairu LN, Pelto GH, Rollins NC, Bland RM, Ntshangase N (2005). Sociocultural influences on infant feeding decisions among HIV-infected women in rural Kwa-Zulu Natal, South Africa. Matern Child Nutr.

[37] Eide M, Myhre M, Lindbæk M, Sundby J, Arimi P, Thior I (2006). Social consequences of HIV-positive women’s participation in prevention of mother-to-child transmission programmes. Patient Educ Couns.

[38] Ostergaard LR, Bula A (2010). “They call our children ”nevirapine babies?” ”: a qualitative study about exclusive breastfeeding among HIV positive mothers in Malawi. Afr J Reprod Health.

[39] Kebaabetswe PM (2007). Barriers to participation in the prevention of mother-to-child HIV transmission program in Gaborone, Botswana a qualitative approach. AIDS Care.

[40] Ministry of Health (2012). Guidelines for Prevention of Mother-to-Child Transmission of HIV/ AIDS in Kenya.

[41] Nachega JB, Morroni C, Zuniga JM, Sherer R, Beyrer C, Solomon S, Schechter M, Rockstroh J (2012). HIV-related stigma, isolation, discrimination, and serostatus disclosure: a global survey of 2035. J Int Ass Physicians in AIDS Care.

[42] Peltzer K, Sikwane E, Majaja M (2011). Factors associated with short-course antiretroviral prophylaxis (dual therapy) adherence for PMTCT in Nkangala district. South Africa Acta Paediatrica.

[43] Peltzer K, Ramlagan S (2011). Perceived stigma among patients receiving antiretroviral therapy: a prospective study in KwaZulu-Natal. South Africa AIDS Care.

[44] Ekama SO, Herbertson EC, Addeh EJ, Gab-Okafor CV, Onwujekwe DI, Tayo F, Ezechi OC (2012). Pattern and determinants of antiretroviral drug adherence among Nigerian pregnant women. J Pregnancy.

[45] O’Gorman DA, Nyirenda LJ, Theobald SJ (2010). Prevention of mother-to-child transmission of HIV infection: views and perceptions about swallowing nevirapine in rural Lilongwe, Malawi. BMC Public Health.

[46] Chinkonde JR, Sundby J, Martinson F (2009). The prevention of mother-to-child HIV transmission programme in Lilongwe, Malawi: why do so many women drop out. Reprod Health Matters.

[47] Church K, Wringe A, Fakudze P, Kikuvi J, Simelane D, Mayhew S, The Integra Initiative (2013). Are integrated HIV services less stigmatising than stand-alone models of care? A comparative case study from Swaziland. J Int AIDS Soc.

[48] Best K (2004). Cambodia: clients find everything they need in one place. Network.

[49] IPPF (2005). Models of Care Project: Linking HIV/AIDS Treatment, Care and Support in Sexual and Reproductive Health Care Settings, Examples in Action.

[50] Maharaj P, Cleland J (2005). Integration of sexual and reproductive health services in KwaZulu-Natal, South Africa. Health Policy Plan.

[51] Topp SM, Chipukuma J, Giganti M, Mwango L, Chiko L, Tambatamba-Chapula B, Wamulume CS, Reid S (2010). Strengthening health systems at facility-level: feasibility of integrating antiretroviral therapy into primary health care services in Lusaka, Zambia. PLoS One.

[52] Ministry of Health (2012). Minimum Package for Reproductive Health (RH) and HIV Integrated Services.

[CR53] The pre-publication history for this paper can be accessed here: http://www.biomedcentral.com/1472-6963/14/412/prepub

